# Local Structural Changes in High-Alumina, Low-Lithium Glass-Ceramics During Crystallization

**DOI:** 10.3390/nano15181449

**Published:** 2025-09-20

**Authors:** Minghan Li, Yan Pan, Shuguang Wei, Yanping Ma, Chuang Dong, Hongxun Hao, Hong Jiang

**Affiliations:** 1 State Key Laboratory of Tropic Ocean Engineering Materials and Materials Evaluation & Special Glass Key Laboratory of Hainan Province, Hainan University, Haikou 570228, China; lmh2169@163.com (M.L.); 23220856010010@hainanu.edu.cn (Y.P.);; 2 School of Ecology, Hainan University, Haikou 570228, China; 3 School of Materials Science and Engineering, Hainan University, Haikou 570228, China; 4 School of Physics and Optoelectronic Engineering, Hainan University, Haikou 570228, China; 5 Key Laboratory of Materials Modification by Laser, Ion and Electron Beams, Dalian University of Technology, Ministry of Education, Dalian 116024, China; dong@dlut.edu.cn; 6 State Key Laboratory of Chemical Engineering, School of Chemical Engineering and Technology, Tianjin University, Tianjin 300072, China; hongxunhao@tju.edu.cn

**Keywords:** glass-ceramics, local structural, crystallization, glass network structure, component aggregation

## Abstract

In this study, we investigate the phase transition process during high-alumina, low-lithium glass-ceramics (ZnO-MgO-Li_2_O-SiO_2_-Al_2_O_3_) crystallization. The differential scanning calorimetry and high-temperature X-ray diffraction results show that approximately 10 wt.% of (Zn, Mg)Al_2_O_4_ crystals precipitated when the heat treatment temperature reached 850 °C, indicating that a large number of nuclei had already formed during the earlier stages of heat treatment. Field emission transmission electron microscopy used to observe the microstructure of glass-ceramics after staged heat treatment revealed that cation migration occurred during the nucleation process. Zn and Mg aggregated around Al to form (Zn, Mg)Al_2_O_4_ nuclei, which provided sites for crystal growth. Moreover, high-valence Zr aggregated outside the glass network, leading to the formation of nanocrystals. Raman spectroscopy analysis of samples at different stages of crystallization revealed that during spinel precipitation, the Q^3^ and Q^4^ structural units in the glass network increased significantly, along with an increase in the number of bridging oxygens. Highly coordinated Al originally present in the network mainly participated in spinel nucleation, effectively suppressing the subsequent formation of Li_x_Al_x_Si_1−x_O_2_, which eventually resulted in the successful preparation of glass-ceramics with (Zn, Mg)Al_2_O_4_ and ZrO_2_ as the main crystalline phases. The grains in this glass-ceramic are all nanocrystals. Its Vickers hardness and flexural strength can reach up to 875 Hv and 350 MPa, respectively, while the visible light transmittance of the glass-ceramic reaches 81.5%. This material shows potential for applications in touchscreen protection, aircraft and high-speed train windshields, and related fields.

## 1. Introduction

Glass-ceramics materials combine the advantages of glass and ceramics, making them an excellent substitute for both materials. They are more cost-effective and easier to prepare than ceramics and offer many unique performance advantages over glass. Moreover, the mechanical, thermal, electrical, and optical properties [1,2] of glass-ceramics can be improved through heat treatment of the base glass [3,4,5,6], even exhibit high-temperature thermal stability [7,8]. As a typical controlled crystallization product, crystalline phase precipitation can be induced in glass-ceramics by dopant elements or nucleating agents, making the composition of the base glass an important factor influencing crystallization. In addition, considering crystals develop from the amorphous base glass, the types of structural units and degree of polymerization in the glass matrix significantly affect both the ease of phase transition and crystallization controllability [9].

The structural features, types, and degree of crystallinity of the precipitated crystals are key factors determining the performance of glass-ceramics [10]. Owing to the difficulty of observing the amorphous state directly and the high-temperature heat treatment required during the crystallization process, studies focusing on the structural changes during crystallization remain limited [11]. To comprehensively investigate the crystallization mechanisms of glass-ceramics, in situ techniques such as high-temperature X-ray diffraction (HT-XRD) are essential for analyzing crystal precipitation, growth, and phase transitions [12]. In addition, analyzing structural changes in the residual glass phase of glass-ceramics provides a more comprehensive understanding of structural evolution during crystallization. At present, spectroscopic methods such as nuclear magnetic resonance (NMR) and Raman spectroscopy are among the most effective techniques for characterizing the structure of amorphous phases [13]. Silicates, regardless of whether they are amorphous or crystalline, are composed of covalently bonded entities as fundamental units. The structural units exhibit characteristic vibrational properties in Raman scattering and molecular dynamics simulations [14,15,16]. These properties facilitate the analysis of the structural features of the crystalline and residual glass phases and enable the modeling of the relationship between their structure and properties.

In their study of the crystallization process, Enriquez et al. [17] successfully obtained information on the ordered and disordered states of the network structure. They used in situ high-temperature Raman spectroscopy to monitor vibrational behavior during crystal precipitation and melting [18] and simulated the corresponding Raman spectra using a plane-wave pseudo-potential method based on density functional theory, with the result indicating reasonable consistency between the experimental data and simulation results. Zhang et al. employed confocal Raman spectroscopy to investigate the effects of adding a nucleating agent (Cr_2_O_3_) on the degree of polymerization in the glass network. Their work contributed to the understanding of the structural transformation from the amorphous to the crystalline states.

In addition to spectroscopic techniques, transmission electron microscopy (TEM) is a crucial tool for analyzing local structural changes during glass-ceramics crystallization [19]. Shen et al. [20] used TEM to observe the migration of silicon groups and cations during crystallization as well as the nucleation and crystal growth processes. They found that omphacite crystals served as nuclei and provided surfaces for crystal growth. Moreover, the consumption of silicon from the glass network during omphacite crystal growth reduced its degree of polymerization and promoted mayenite crystallization. Zhou et al. [21] developed a novel Si_3_N_4_-SiO_2_ glass-ceramics and used scanning transmission electron microscopy to examine its morphology and microstructure and investigate the influence of its crystalline and residual glass phase compositions on its properties. They observed intergranular glass films (IGFs) with thicknesses of several nanometers between the grains. Based on results from prior studies [22], they concluded that the formation of IGFs is related to the interfacial energy between the grains. Beibei Ren et al. [23] utilized characterization techniques such as FTIR, NMR, and XPS to analyze and confirm that the crystallinity increases with rising heat treatment temperature. The proportion of bridging oxygens (BO) in the residual glass phase rises significantly, while the proportion of non-bridging oxygens (NBO) decreases, indicating an enhanced polymerization degree of the glass network. Jianghai Huang et al. [24] explored the nucleation mechanism in glass-ceramics using FTIR and XRD. Their results demonstrate that ZrO_2_ can provide heterogeneous nucleation sites, promoting the crystallization of spinel (MgAl_2_O_4_). SnO_2_ dissolves into the glass network in the form of [SnO_4_] tetrahedra and precipitates as SnO_2_ crystals at high temperatures, simultaneously acting as a fining agent. The combined use of both can increase nucleation density and inhibit excessive grain growth. Furthermore, Dr. Shichalin’s team [25,26] employed characterization methods like EXAFS to analyze the short-range structure of amorphous materials. This approach allows for a clear determination of changes in the local coordination environment of amorphous materials and also provides new insights for characterizing structural changes in glass-ceramics before and after crystallization [27,28]. ZnO-MgO-Li_2_O-SiO_2_-Al_2_O_3_ glass-ceramics with spinel as the primary crystalline phase exhibit excellent mechanical properties (Vickers hardness exceeding 950 Hv; flexural strength above 350 MPa) [29].

Simultaneously, due to the small size of the precipitated crystals, these glass-ceramics possess relatively high transparency compared to other spinel ceramics or glass-ceramics [1,30,31]. Consequently, owing to their combination of superior mechanical properties and transmittance, they can serve as cover glass for electronic device touchscreens and protective glass for deep-sea exploration equipment, enabling operation in complex environments. Moreover, compared to other glass-ceramic systems, they feature higher melting and crystallization temperatures. Research concerning their nucleation and crystal growth mechanisms during the crystallization process remains relatively scarce. Compared with other glass-ceramics systems, ZnO-MgO-Li_2_O-SiO_2_-Al_2_O_3_ glass-ceramics has higher melting and crystallization temperatures. However, its nucleation and crystal growth mechanisms have not yet been extensively studied. Therefore, this study aims to investigate the effects of local structural changes during the crystallization process of ZnO-MgO-Li_2_O-SiO_2_-Al_2_O_3_ glass-ceramics. The precipitation of multiple crystalline phases was analyzed using HT-XRD. The glass-ceramics samples with varying degrees of crystallization were prepared by applying different heat treatment processes to the base glass. The structural evolution of the amorphous phase during crystallization was clarified by analyzing the changes in the Q^n^ species in both the base and residual glass phases of the glass-ceramics. The phase separation mechanism during nucleation was investigated by combining Raman spectroscopy analysis with high-resolution TEM (HR-TEM), with the crystal formation and growth processes elucidated. A previous study [22] reported that highly coordinated Al exists in the glass network and promotes the growth of (Zn, Mg)Al_2_O_4_. Considering the content of the Q^n^ units in the residual glass phase necessarily changes in regions where spinel precipitates, analyzing the changes in Q^n^ species within the glass network would help reveal how the amorphous phase transforms into a crystalline phase and, consequently, infer the nucleation and crystal growth modes. This study aims to investigate the nucleation mechanisms of several crystalline phases. By employing techniques such as Raman spectroscopy, changes in Q^n^ species within high-alumina low-lithium base glasses and nucleated glasses were characterized to analyze structural evolution in the amorphous phase. Based on this, the formation and growth pathways of crystals in the glass-ceramics were deduced. Ultimately, the influence of crystal type, crystallinity, and other factors on the structure and properties of the glass-ceramics is elucidated.

## 2. Experimental

The composition of the ZnO-MgO-Li_2_O-SiO_2_-Al_2_O_3_ base glass used in this study is listed in Table 1. In this study, raw materials such as silica sand, Al_2_O_3_, ZrO_2_, MgO, Na_2_CO_3_, and Li_2_CO_3_ were employed to introduce the various compositions listed in Table 1. The main raw materials were melted at 1650 °C for 1.5 h, poured into preheated stainless steel molds, and annealed at 600 °C for 4 h. The resulting base glass and the corresponding nucleated and glass-ceramics were designated as BG. Differential Scanning Calorimetry (DSC) of the base glass was performed using a simultaneous thermal analyzer (STA 509 Jupiter Classi, NETZSCH, Selb, Germany). Three small samples collected from different regions of the base glass were ground into powder and tested separately. The tests were conducted over a temperature range of 30~1400 °C at a heating rate of 10 °C/min under a N_2_ atmosphere. The corresponding nucleated glass and glass-ceramics are designated as HG and CG, respectively, with their formulation listed in Table 1.

Glass sheets were cut to dimensions of 20 × 20 × 1 mm and polished using a polishing machine. Variable-temperature X-ray diffraction measurements were performed using a Bruker D8 Advance diffractometer (Bruker, Ettlingen, Germany). The temperature was increased from room temperature, with a scan performed at 30 °C increments from 700 °C up to 1100 °C, at which point the final scan was completed.

The Raman spectra of the base and glass-ceramics from 200 to 2000 cm^−1^ were measured using a Raman spectrometer (Horiba LabRAM HR Evolution, Kyoto, Japan) with a 532 nm excitation source. For each sample, three separate samplings were performed (approximately 7 mg per sampling), with three replicate measurements conducted per sampling to acquire the Raman spectra. Raman spectra were deconvoluted using Gaussian functions in PeakFit 4.1.2 software. The fitting range was selected as 850~1250 cm^−1^, baselines were corrected with the Progressive Lin algorithm, and three independent deconvolution operations were performed for each spectrum to ensure reproducibility.

The microstructures of the base and glass-ceramics were examined using field emission transmission electron microscopy (FE-TEM, Thermo Scientific Talos F200X G2, Waltham, Ma, USA), with their elemental distributions analyzed using energy-dispersive spectroscopy (EDS). For both glass and glass-ceramic samples, three small fragments were selected from different regions and ground into powder. The crushed powders were individually dispersed in anhydrous ethanol via ultrasonication. A 50 μL aliquot of the supernatant from each sample was drop-cast onto a carbon support film and dried to prepare corresponding FE-TEM specimens. During analysis, five distinct areas within each specimen were selected for high-resolution imaging and EDS characterization [32].

The optical transmittance of both the base glass and crystallized samples within the wavelength range of 300–800 nm was characterized at room temperature using a PerkinElmer Lambda 750s ultraviolet-visible spectrophotometer (Waltham, MA, USA). All specimens were prepared with a consistent thickness of 2.5 mm. Prior to mechanical testing, each sample—including both base glass and glass–ceramics—was polished to achieve parallel surfaces of uniform 2.5 mm thickness. Bend tests were performed on a Yuhan YC-128A universal testing machine (Shanghai, China), and Vickers hardness was measured using a Kejing UNIPOL-802 hardness tester (Shenyang, China).

## 3. Results and Discussion

### 3.1. Differential Scanning Calorimetry (DSC)

**Figure 1:**
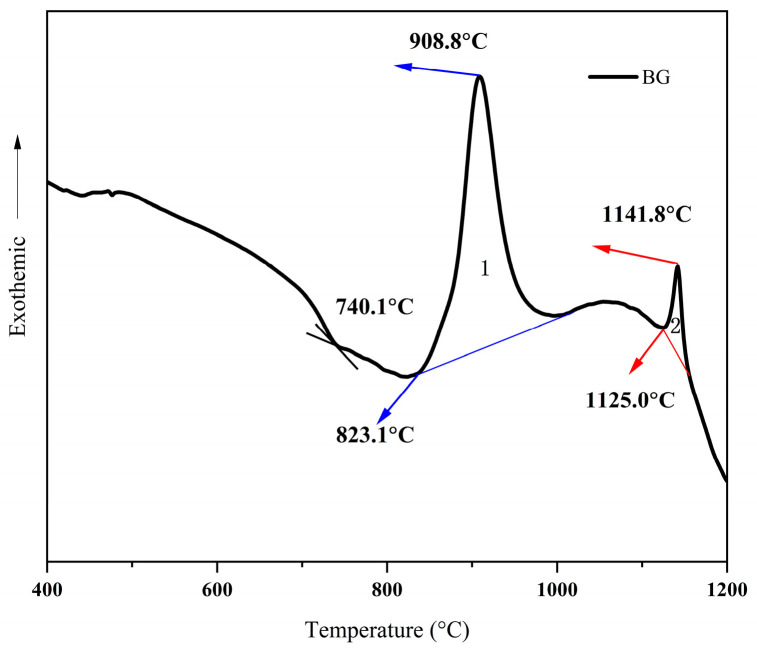
DSC curve of base glass sample.

The characteristic temperatures observed in the differential scanning calorimetry curves obtained from three independent DSC tests of the base glass samples showed close agreement. The variation in characteristic temperatures across the three measurements was less than 0.3%, which does not affect the selection of subsequent crystallization processes. Hence, one representative sample was selected for further analysis. Figure 1 shows the DSC curve of the base glass. The presence of two independent crystallization peaks in the BG sample indicates that more than one crystalline phase may have precipitated or a phase transition may have occurred during heat treatment. The onset temperatures of the two crystallization peaks are approximately 823.1 °C and 1125.0 °C, respectively, and the glass transition temperature is 740.1 °C. The DSC curve analysis was performed using TA Universal Analysis 2000 and Origin 2021 software, yielding crystallization enthalpies of 35.8 J/g for Peak 1 and 6.7 J/g for Peak 2.

### 3.2. HT-XRD

**Figure 2:**
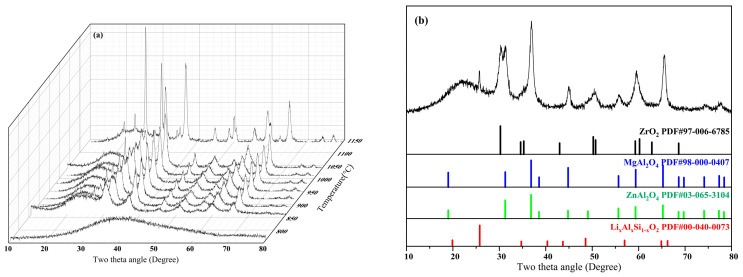
(**a**,**b**) In situ XRD spectrum.

To further investigate the crystallization behavior of polycrystalline-phase glass-ceramics, in situ XRD was employed to analyze the crystalline phases, their precipitation sequence, and the degree of crystallinity (Figure 2). At test temperatures below 830 °C, no crystals precipitated from the base glass. As the temperature increased to 850 °C, crystallization peaks began to appear, indicating that the (Zn, Mg)Al_2_O_4_ solid solution phase precipitated first. The interplanar spacing calculated from the crystallization peak at 31.30° (2θ) in the XRD pattern is 2.86 Å, corresponding to the (220) planes of both ZnAl_2_O_4_ and MgAl_2_O_4_ (d(220) = 2.86 Å for both). Given the simultaneous presence of Zn and Mg in the base glass composition, coupled with their compatible crystal structures and matched ionic radii, these components readily form a solid solution. We therefore confirm that the crystalline phase precipitated after heat treatment is the (Zn, Mg)Al_2_O_4_ solid solution.

Upon further heating to 910 °C, the precipitation of (Zn, Mg)Al_2_O_4_ progressively increased without formation of additional crystalline phases, consistent with the DSC results. When the temperature exceeded 950 °C, ZrO_2_ and Li_x_Al_x_Si_1−x_O_2_ crystalline phases began precipitating. Notably, their precipitation temperatures were lower than the DSC peak onset temperatures. This phenomenon occurs because the addition of the nucleating agent ZrO_2_ reduces the energy barrier required for nucleation. Consequently, various crystalline phases nucleate at lower temperatures, while subsequent heating provides energy for crystal growth, enabling earlier precipitation of ZrO_2_ and Li_x_Al_x_Si_1−x_O_2_ phases. At 1120 °C, the overall crystallinity of the glass-ceramic exhibited a significant increase, resulting in an opaque appearance due to light scattering.

(Zn, Mg)Al_2_O_4_ constitutes a solid solution of ZnAl_2_O_4_ and MgAl_2_O_4_, corresponding to PDF#98-000-0407 (MgAl_2_O_4_) and PDF#03-065-3104 (ZnAl_2_O_4_), respectively. Its cubic unit cell parameter is a = 8.07429 Å. Li_x_Al_x_Si_1−x_O_2_ is indexed to PDF#00-040-0073 with orthorhombic unit cell parameters: a = 9.51851 Å, b = 8.48999 Å, c = 5.31593 Å. ZrO_2_ (PDF#97-006-6785) exhibits a tetragonal phase (t-ZrO_2_) as determined by its d(110) = 2.54 Å. The unit cell belongs to the tetragonal system (a = b ≠ c, α = β = γ = 90°) with refined parameters: a = b = 3.60498Å, c = 5.21 Å.

To further validate the structural accuracy, Rietveld whole-pattern fitting refinement was performed on the XRD patterns of the glass-ceramic using GSAS-II 5585 software. The background was modeled with a Chebyshev function, while refinement parameters included lattice constants, instrument parameters, atomic coordinates, and thermal displacement parameters to achieve optimal agreement between the simulated and experimental diffraction patterns. The converged refinement provided quantitative phase analysis and precise lattice parameter determination. For the sample crystallized at 1120 °C, the overall crystallinity at this stage is approximately 40 wt%. Meanwhile, the phase proportions of (Zn, Mg)Al_2_O_4_, ZrO_2_, and Li_x_Al_x_Si_1−x_O_2_ constitute 16.8 wt%, 10.0 wt%, and 73.2 wt%, respectively. The results demonstrate excellent agreement between the calculated and experimental patterns across the entire angular range (reliability factors: Rwp = 10.807%, χ^2^ = 4.71).

### 3.3. TEM-EDS

**Figure 3:**
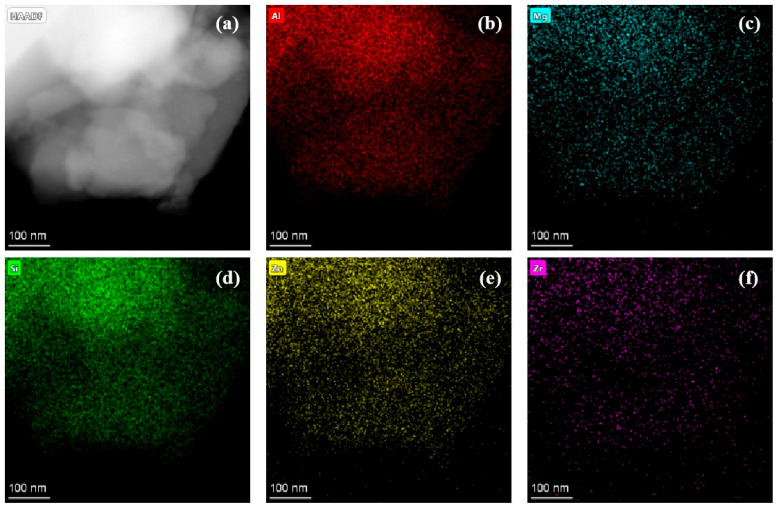
TEM-EDS diagram of base glass. (**a**) HR-TEM images of the BG samples; (**b**–**f**) High-angle annular dark field (HAADF) image and element distribution of the BG samples.

TEM-EDS was performed on the ZnO-MgO-Li_2_O-SiO_2_-Al_2_O_3_ base and nucleated glasses. The elemental maps shown in Figure 3 indicate the elements are uniformly distributed in the bulk of the base glass, with their composition consistent with that listed in Table 1. TEM-EDS analysis revealed that the weight percentages (wt%) of five key elements—Al, Mg, Si, Zn, and Zr—were 35.8%, 5.8%, 43.9%, 10.5%, and 4.0%, respectively. These measured values closely match the designed composition.

To directly visualize the crystallization process of the base glass, FETEM was conducted on heat-treated samples to observe their microstructural features during glass-ceramics nucleation.

**Figure 4:**
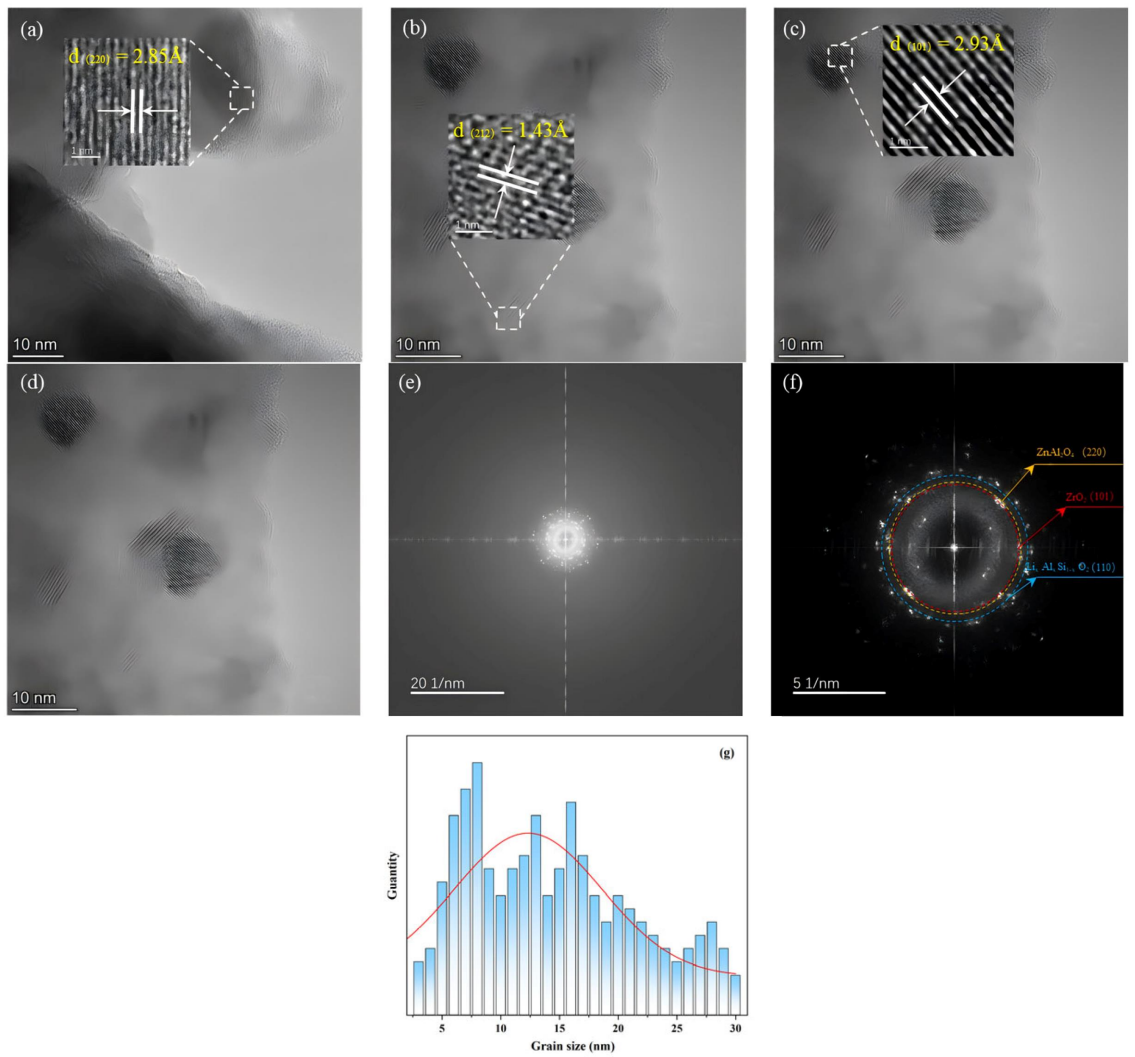
(**a**–**d**) HRTEM images of GC3 samples.(**e**,**f**) High-angle annular dark field images of crystals. (**g**) Grain Size Distribution Statistics.

The DSC and HT-XRD results suggest that base glass nucleation likely occurred in the temperature range of 760–820 °C. However, the nuclei formed within the glass matrix for detection may have been insufficient. Therefore, FETEM was used to analyze the microstructure and verify nucleation at this stage. As shown in Figure 4, nanocrystals formed after heat treatment of the base glass at 790 °C for 6 h, providing nucleation sites for subsequent crystal growth. Figure 4a–d show FETEM images of the base glass after heat treatment at 790 °C for 6 h. Ten lattice planes in each of the three regions were measured to determine the crystal interplanar spacing. The obtained interplanar spacings at positions in Figure 4a–c are 0.285, 0.143, and 0.255 nm, respectively. Compared with standard XRD patterns, The measured interplanar spacing of 0.286 nm corresponds to the (220) planes of ZnAl_2_O_4_ and MgAl_2_O_4_ crystals (d(220) = 2.86 Å). EDS analysis confirming the co-distribution of Zn and Mg elements within the crystallization regions further supports the identification of the precipitated phase as (Zn, Mg)Al_2_O_4_ solid solution; The 0.143 nm spacing to the (212) plane of Li_x_Al_x_Si_1-x_O_2_ phase (d(212) = 1.43 Å), and the 0.255 nm spacing to the (110) plane of ZrO_2_ crystal (d(110) = 2.54 Å).

During the nucleation of (Zn, Mg)Al_2_O_4_, cation migration occurred, and Zn and Mg aggregated at sites with highly coordinated Al to form a (Zn, Mg)Al_2_O_4_ solid solution and initiate crystal growth, as inferred from Figure 4a,d. Before heat treatment, various cations and structural groups were uniformly distributed throughout the base glass. After heat treatment, Al groups aggregated and formed Al-rich regions, allowing cations such as Zn and Mg to contact Al more easily and form (Zn, Mg)Al_2_O_4_ solid solution nanocrystals, whose growth was promoted by energy supplied during heat treatment.

Owing to the sufficient ZrO_2_ content in the base glass composition, Zr exhibits high coordination and exists outside the glass network as [ZrO_8_] units. The tendency of these units to segregate leads to the formation of Zr-rich heterogeneous regions [33]. The ongoing heat treatment continuously supplied energy for nucleation, resulting in the precipitation of ZrO_2_ nanocrystals. As shown in Figure 4d, a certain degree of Al and Zn aggregation occurred around the Zr groups. This aggregation resulted in a diffusion barrier composed of Al and Zn around the Zr groups, which hindered Zr diffusion [34] and thereby limited the growth of ZrO_2_ crystals. Therefore, all the ZrO_2_ crystals were nanocrystalline.

### 3.4. Raman Spectroscopy

According to silicate structure theory [35,36], oxygen in the glass network exists in two forms: bridging and non-bridging oxygens. Their ratio influences the network structure of glass and, consequently, its properties. The glass network contains various types of silicon-oxygen tetrahedral structural units. Each unit is associated with a different number of bridging oxygens. The notation Q^n^, where n represents the number of bridging oxygens, is introduced to describe the types of structural units in the glass. Q^n^ denotes the average number of bridging oxygen atoms per silicon-oxygen tetrahedron. The Raman spectroscopy wavenumber range of 800–1200 cm^−1^ in silicate glass melt structures corresponds to the symmetric and antisymmetric stretching vibrations of bridging and non-bridging oxygens. The correspondence between the Q^n^ units and the wavenumbers and vibrational modes is summarized in Table 2.

**Figure 5:**
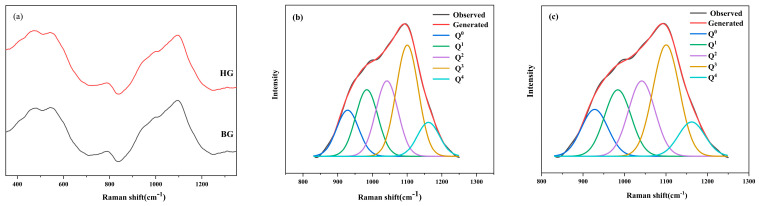
(**a**–**c**) Raman spectra of base and nucleated glasses (BG, HG) and their corresponding spectral fits.

The Raman spectra of the base glass in the ZnO-MgO-Li_2_O-SiO_2_-Al_2_O_3_ system are shown in Figure 5. Three important vibrational bands are observed from the Raman spectroscopy results in the 200–1300 cm^−1^ range [39]: the band at approximately 500 cm^−1^ corresponds to the overall bending vibration of the Si-O bonds; a weaker band at approximately 700 cm^−1^ is attributed to the tetrahedral structure (Q^0^); the band near 1000 cm^−1^ corresponds to Si-O stretching vibrations (n(Q^n^)).

The base glass was subjected to crystallization under different processes and analyzed using Raman spectroscopy. First, the nucleated glass HG was prepared through heat treatment at a nucleation temperature of 760 °C for 4 h. Subsequently, glass-ceramic samples CG1~CG4 were produced by employing the same nucleation parameters (760 °C/4 h) followed by crystallization treatments at 860, 920, 980, and 1040 °C, respectively, each maintained for 1 h. The fitted curves of the Raman spectra from 800 to 1300 cm^−1^ for the samples with various degrees of crystallinity are shown in Figure 5b,c. The changes in the glass network were evaluated using the ratio of non-bridging oxygens (NBO/NBO + BO), average number of non-bridging oxygens per tetrahedron (NBO/tetrahedron), and average number of bridging oxygens per tetrahedron (bridges/tetrahedron):

(1) $$\frac{\mathrm{NBO}}{\mathrm{NBO}+\mathrm{BO}}=\frac{\sum Q^{n}(4-n)}{\sum O},$$

(2) $$\frac{\mathrm{NBO}}{\mathrm{tetrahedron}}=\frac{\sum Q^{n}(4-n)}{\left[\mathrm{Si}+\mathrm{Al}\right]},$$

(3) $$\frac{\mathrm{bridges}}{\mathrm{tetrahedron}}=\frac{\sum n}{\left[\mathrm{Si}+\mathrm{Al}\right]}.$$

The structural evolution of the glass network during the crystallization process of ZnO-MgO-Li_2_O-SiO_2_-Al_2_O_3_ glass was analyzed. The calculated parameters characterizing the network structure for un-crystallized and nucleated glass and the various crystallization stages are summarized in Table 3.

Figure 5a–c shows the distribution of Q^n^ structural units in the base and nucleated glasses. As heat treatment progressed, the content of Q^0^ and Q^1^ decreased in the nucleated glass while those of Q^3^ and Q^4^ increased. In contrast, the average number of non-bridging oxygens per tetrahedron (NBO/tetrahedron) decreased from 1.94 to 1.89. This result suggests that during the nucleation of crystalline phases such as (Zn, Mg)Al_2_O_4_, cations bound to non-bridging oxygens in the glass network, thereby increasing the degree of network connectivity and forming a more integrated structure [40,41].

Based on the bonding energy between the cations and oxygen, oxides in the glass network can be classified into three types [36,39] comprising (1) network formers, which constitute the glass network structure and impart stability, enabling glass formation; (2) network modifiers, which cannot form glass on their own and disrupt the glass network; (3) network intermediates, which exhibit behavior intermediate between those of network formers and modifiers [41]. In the base glass of the system, Al_2_O_3_ exists partly in the form of tetrahedral [AlO_4_] units, which act as network formers, and partly as ^V^Al, which functions as a network intermediate [20]. Upon heat treatment, ^V^Al binds with free oxygen to form ^VI^Al, which contributes to the increase in Q^4^ content in the network.

**Figure 6:**
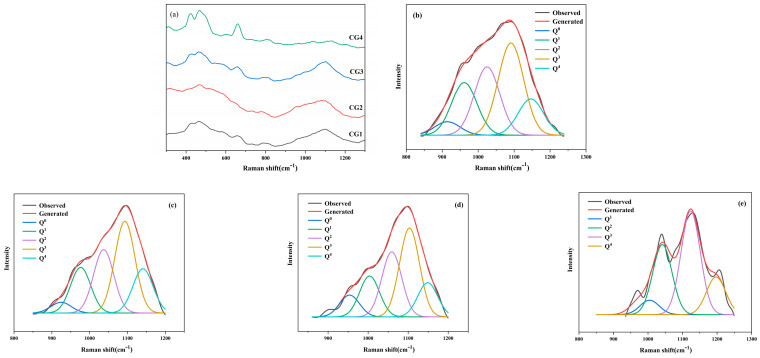
(**a**–**e**) Raman spectra and fitted Raman curves of glass-ceramics at different heat treatment stages (CG1–CG4).

High-field-strength Zr ions outside the glass network also influence the network structure if such ions are present. The Raman spectra and Gaussian multi-band fitting results (Figure 6 and Table 3) combined with the various experimental data indicate that (Zn, Mg)Al_2_O_4_ spinel precipitated first as the crystallization temperature increased. At this stage, the Q^0^ and Q^1^ content decreased while those of Q^3^ and Q^4^ increased. ZrO_2_ crystals precipitated subsequently, further increasing the degree of glass network polymerization, as indicated by the significant rise in the proportion of Q^3^ and Q^4^ units.

Comparing Figure 5 and Figure 6 and referring to Table 3, crystallinity increased significantly with the heat treatment temperature. Q^0^ and Q^1^ units gradually transformed into Q^3^ and Q^4^ units. At the same time, the number of non-bridging oxygens in each tetrahedron (NBO/tetrahedron) decreased from 1.73 to 1.34. This change is attributed to the binding of ^V^Al with free oxygen during heat treatment, which promoted the nucleation and subsequent separation of ^V^Al from the glass network, thereby increasing network polymerization. During the heat treatment process, heterogeneous nucleation sites for subsequent crystallization formed. When the temperature reached the glass transition temperature T_g_, ^V^Al bound with oxygen, while Zn and Mg aggregated in the vicinity, resulting in the polarization and phase separation of certain components [42,43]. After heat treatment, the increased polymerization of the glass network promoted nucleation and crystallization, leading to the formation of microcrystalline phases.

**Figure 7:**
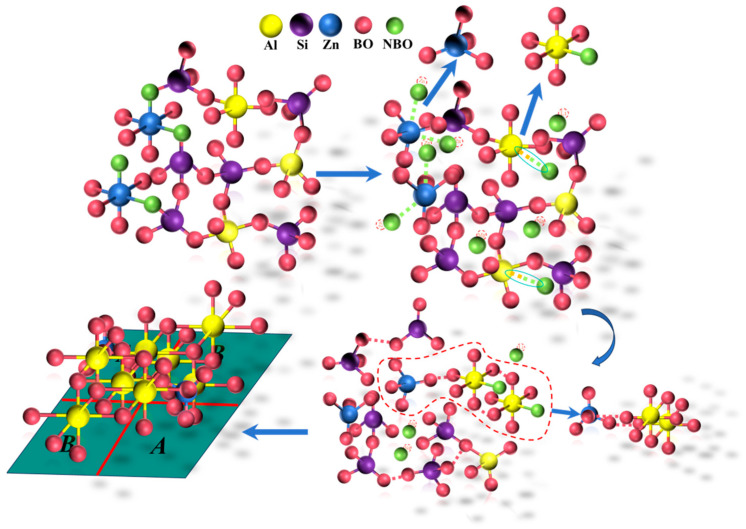
Schematic diagram of local structural changes during crystallization.

Figure 7 shows the local structural changes in the glass phase during the crystallization process. Owing to the high Zn content in the base glass, Zn existed in a highly coordinated state within the glass network. Compared to covalent bonds in network formers, ionic bonds formed through electrostatic attraction are more easily broken. Zn-O-Si linkages in the glass network contain ionic Zn-O bonds and covalent Si-O bonds. These mixed-bond configurations facilitated rapid bond breakage during crystallization, promoting the formation of zinc-oxygen tetrahedra in the spinel structure. As the temperature increased, ^V^Al bound with free oxygen and promoted the formation of surrounding ^VI^Al, which then combined with zinc tetrahedra to form the AB structure of (Zn, Mg)Al_2_O_4_. Meanwhile, broken Si-O tetrahedra gradually reconnected with Al-O tetrahedra to form Si-O-Si and Si-O-Al linkages, resulting in a denser glass network structure.

**Figure 8:**
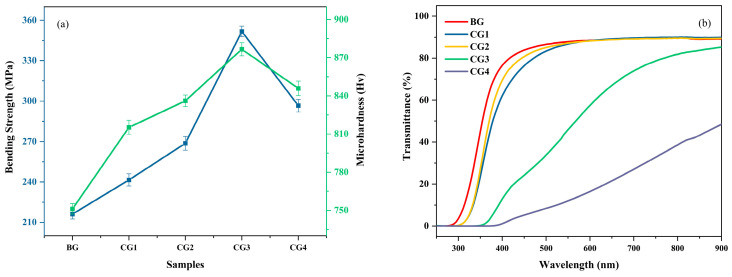
(**a**) Flexural strength and microhardness of the base glass and glass-ceramic samples; (**b**) Visible light transmittance of the base glass and glass-ceramic samples.

Figure 8a shows the flexural strength and microhardness of the base glass (BG) and glass-ceramic samples (CG1–CG4), clearly illustrating the changes in Vickers hardness and flexural strength before and after crystallization. The Vickers hardness and flexural strength of the base glass are approximately 750 Hv and 210 MPa, respectively. As the heat treatment temperature increases, the crystallinity of the glass-ceramics rises, leading to a significant improvement in mechanical properties. The maximum Vickers hardness and flexural strength reach 875 Hv and 350 MPa, respectively. Based on HT-XRD results, from sample CG1 to CG3, the enhancement is primarily attributed to the increased precipitation of (Zn, Mg)Al_2_O_4_. The nano-sized (Zn, Mg)Al_2_O_4_ crystals contribute to higher hardness and inhibit crack propagation, resulting in notable improvements in both hardness and flexural strength. However, with a further increase in heat treatment temperature, Li_x_Al_x_Si_1-x_O_2_ begins to precipitate extensively, agglomerating on the spinel crystals and leading to increased crystal size. Under higher applied stress, the weaker bonding strength of the quartz solid solution facilitates crack initiation at these junctions, resulting in a decline in mechanical performance.

Figure 8b shows that the transmittance of the base glass (BG) before crystallization exceeds 90%. The transmittance of the glass-ceramics near 800 nm wavelength is 90%, 89.5%, 81.5%, and 39%, respectively. Due to crystal precipitation, the transmittance of the glass-ceramics slightly decreases compared to the base glass. Samples CG1 to CG3 maintain high transparency with transmittance above 85%, whereas sample CG4 exhibits a significant reduction in transmittance owing to the extensive precipitation of Li_x_Al_x_Si_1-x_O_2_, which agglomerates around the spinel and increases crystal size.

## 4. Conclusions

Through in situ XRD analysis, it was determined that during the crystallization of the ZnO–MgO–Li_2_O–SiO_2_–Al_2_O_3_ base glass, when the heat treatment temperature was below 1000 °C, (Zn, Mg)Al_2_O_4_ precipitated first, while ZrO_2_ and a small amount of Li_x_Al_x_Si_1−x_O_2_ precipitated during subsequent heat treatment stages. TEM analysis of the (Zn, Mg)Al_2_O_4_ nucleation process revealed that Zn and Mg aggregated around Al to form (Zn, Mg)Al_2_O_4_ nanocrystals, which precipitated preferentially. A small portion of the remaining Al ions combined with Si and Li in the glass network to form Li_x_Al_x_Si_1−x_O_2_. These results suggest that the formation of (Zn, Mg)Al_2_O_4_ inhibited the nucleation and crystallization of Li_x_Al_x_Si_1−x_O_2_. Owing to its high-valence state and refractory nature, ZrO_2_ formed localized fields and precipitated independently during the heat treatment process. However, as the heat treatment temperature increases, the necessary energy is provided for the growth of the Li_x_Al_x_Si_1−x_O_2_ crystalline phase, enabling it to precipitate earlier and undergo sustained growth. Under these conditions, the small amount of (Zn, Mg)Al_2_O_4_ that precipitated initially is no longer able to inhibit the nucleation and growth of Li_x_Al_x_Si_1−x_O_2_.

Moreover, to reveal the structural changes in glass-ceramics during the crystallization process, the characteristic vibrational modes of the glass network were analyzed using Raman spectroscopy. The Q^n^ distribution revealed by Raman mapping clarified the structural evolution of the glass network. The data obtained from band fitting further indicated the formation of bridging oxygens and polymerization in the glass network. Raman analysis of glass-ceramics at different stages of crystallization showed that as the heat treatment temperature increased, high-valence Al (^V^Al) bonded with free oxygen, leading to a significant decrease in Q^0^ and Q^1^ units in the glass network. Additionally, in the regions where spinel precipitated, Q^3^ and Q^4^ units increased significantly, indicating that nucleation occurred preferentially at these sites. With continued heat treatment, (Zn, Mg)Al_2_O_4_ spinel nuclei grew into nanocrystals. Performance analysis of the glass-ceramics indicates that under the heat treatment process of 780 °C/4 h + 980 °C/1 h, the Vickers hardness and flexural strength can reach up to 875 Hv and 350 MPa, respectively, with a visible light transmittance of 81.5%. This suggests potential for applications in various fields.

## Figures and Tables

**Table 1:** Composition of base glass (wt.%).

wt%	SiO_2_	Al_2_O_3_	ZnO	MgO	ZrO_2_	Li_2_O	Na_2_O
BG	40.70 ± 0.05	33.90 ± 0.03	11.20 ± 0.03	5.50 ± 0.03	4.5 ± 0.02	2.70 + 0.02	1.5 ± 0.02

**Table 2:** Characteristic vibrations of bridging oxygens [37,38].

Q^n^	Bands (cm^−1^)	Recommended Assignments	Structural Unit
Q^0^	870	The Si-O band stretching vibration in fully non-bridging oxygen tetrahedra	[SiO_4_]^4−^
Q^1^	905	the Si-O band stretching vibrations in tetrahedra with three non-bridge oxygen per tetrahedron	[Si_2_O_7_]^6−^
Q^2^	960	the Si-O band stretching vibrations in tetrahedra with two non-bridge oxygen per tetrahedro	[SiO_3_]^2−^
Q^3^	1015	the Si-O band stretching vibrations in tetrahedra with one non-bridge oxygen per tetrahedron	[Si_2_O_5_]^2−^
Q^4^	1060	the Si-O band stretching vibration in fully polymerized units	SiO_2_

**Table 3:** Raman parameters of glass and nucleated glass.

	BG	HG	CG1	CG2	CG3	CG4
SQ^0^	13.87 ± 3%	13.05 ± 3%	7.97 ± 3%	6.14 ± 3%	5.99 ± 3%	0.00%
SQ^1^	20.25 ± 3%	19.19 ± 3%	19.15 ± 3%	17.78 ± 3%	16.29 ± 3%	10.01 ± 3%
SQ^2^	22.78 ± 3%	22.76 ± 3%	24.67 ± 3%	24.82 ± 3%	25.92 ± 3%	31.16 ± 3%
SQ^3^	32.60 ± 3%	33.32 ± 3%	33.90 ± 3%	34.95 ± 3%	35.45 ± 3%	41.16 ± 3%
SQ^4^	10.51 ± 3%	11.67 ± 3%	15.20 ± 3%	16.32 ± 3%	16.35 ± 3%	17.79 ± 3%
NBO/(NBO + BO)	0.94 ± 0.08	0.90 ± 0.08	0.76 ± 0.09	0.68 ± 0.10	0.67 ± 0.10	0.50 ± 0.10
NBO/tetrahedron	1.94 ± 0.09	1.89 ± 0.09	1.73 ± 0.10	1.62 ± 0.11	1.60 ± 0.11	1.34 ± 0.12
bridges/tetrahedron	2.06 ± 0.09	2.11 ± 0.09	2.27 ± 0.10	2.38 ± 0.11	2.40 ± 0.11	2.66 ± 0.12

## Data Availability

Data is contained within the article.
